# Case of Imperforate Hymen, Occasioning Retention of the Menstrual Fluid—Division of the Membrane, &c.

**Published:** 1839-10-26

**Authors:** James F. Latta

**Affiliations:** Chester County, Pa.


### MEDICAL EXAMINER.

DEVOTED TO MEDICINE, SURGERY, AND THE COLLATERAL SCIENCES.

No. 43.] PHILADELPHIA, SATURDAY, OCTOBER 26, 1839. [Vol. II.

### CASE OF IMPERFORATE HYMEN, *occasioning retention of the menstrual fluid—Division of the membrane, &c.* By James F. Latta, M. D.

I was called, on the 8th of June last, to see Miss ——, a young lady nearly fifteen years of age, whose case presented the following symptoms:—Slight fever; distention and hardness of the abdomen, increasing in the direction of the pubes; violent pain in the hypogastric region, with frequent *paroxysmal* aggravations; *entire suppression of urine.*

On making some inquiry with regard to the previous health of my patient, I was told that she had always been considered of a delicate constitution; that she had never menstruated, and that she had once, about six months previously, suffered from symptoms similar to those (though less intense) for which I was now called to prescribe.

My views of the case were, that the constitutional symptoms and pain were occasioned by the accumulation of urine in the bladder, giving rise to inordinate distention, and, perhaps, incipient inflammation of its coats. The cause of the suppression I presumed to be some irritation of the muscular tissue contributing to the formation of the *cervix vesicæ*, so that the stimulus of every urinous effort would excite spasmodic contraction of the sphincter.

To meet the indications based upon this diagnosis, bleedings, fomentations, camphor, and opium, and the warm bath, were severally employed, without producing any other effect than temporary relief from pain. The suppression continued. I now returned to my office, and procured a catheter, by means of which a large quantity of urine was evacuated. Alleviation of all the symptoms followed immediately, and my patient appeared to convalesce rapidly, enjoying entire exemption from pain—while the bladder experienced no further difficulty in the discharge of its functions. In the course of a few days, however, when attempting to sit up, a severe, dragging pain was felt in the lumbar region, extending to the hips, and accompanied by an irresistible bearing down effort. On resuming the recumbent position, all these symptoms disappeared. Here were readily recognised the more characteristic evidences of *prolapsus uteri.* My attention was also directed to a *tumour* of considerable dimensions, occupying the hypogastric and part of the umbilical regions, which, upon examination, communicated to the hand much the same impression conveyed by the uterine globe, in most cases, a few days after delivery. Every attempt to continue the sitting posture was attended by the distressing sensations above described. I directed the girl, by uniting her hands and raising, as well as she could, the abdominal viscera, to imitate the action of the utero-abdominal supporter, whose merits have been recently set forth by your intelligent correspondent and my skilful preceptor, Dr. William Harris. This manipulation afforded very decided relief. As soon, however, as it was discontinued, the pain returned.

Conceiving that the symptoms of prolapsus were occasioned by the tumour referred to—whatever it *might* be,—and driven, after many surmises, to the conclusion that it *must* be chronic enlargement of the bladder, I directed a decoction of uva ursi, with a plaster of Burgundy pitch to the loins. Under this treatment—without any diminution in the size of the tumour—my patient grew rapidly better, and in a few days I took my leave.

On the 7th July—after the lapse of one catamenial period, it will be observed—I was again called to combat, in the person of the same individual, the symptoms I had encountered before. Without using any preliminary measures, I introduced the catheter at once, and evacuated the urine. After withdrawing the instrument, it was thought advisable to take advantage of the opportunity, to ascertain the condition of the uterus. Passing my finger between the labia, I found them partially separated by a membrane protruding almost to a level with their external surface, which yielded, upon pressure, a sense of fluctuation, being apparently pressed forward by a fluid accumulated behind it. Further examination led to the discovery that, in the person of this young lady, existed the rare phenomenon of an *imperforate hymen.*

I say *rare*, because I am not aware that a case of the kind has ever been reported in this country; and few are recorded by medical writers, whether here or elsewhere. The enlarged opportunities of Dr. Dewees have never presented him with a single case.

All my speculations with regard to the tumour were now at rest. It could be none other than the uterus itself, distended by the accumulated product of its own secreting vessels. The suppression of urine was, doubtless, occasioned by mechanical pressure of the menstrual fluid upon the *cervix vesicæ.*

That my own views might be confirmed, as well as to extend the opportunity of witnessing a rare and interesting case, I proposed that my friend Dr. B. R. Erwin should be called in, which was accordingly done. After careful examination, Dr. E. expressed his opinion as entirely concurrent with mine,—whereupon we decided upon dividing the membrane immediately.

For this purpose, after placing the patient conveniently for the operation, I took in my hand a common scalpel, with which I was obliged to make very firm pressure, before the resistance offered could be overcome—the instrument appearing to contend with a ligamentous tissue, rather than the simple mucous membrane which ordinarily constitutes the hymen. The passage of the knife being effected, and the extent of the incision prolonged, our expectations were realized by the gushing forth of the long-obstructed catamenia.

The quantity of fluid discharged at this time did not exceed a pint. On the following morning I injected into the vagina a little tepid water, when the flow was re-established, continuing longer and in larger quantity than before. During the remainder of this day, after the patient was put to bed, the fluid continued to pass from her to the amount, perhaps, of half a pint. On the succeeding day, June 9th, the flow was suppressed; tenderness in the hypogastric region was complained of. On the 10th *hysteritis* was fully developed. For some days the young lady's life was despaired of; the more acute symptoms yielded, however, to bleeding, purging, warm fomentations, &c. After the subsidence of the fever, a discharge from the uterus followed, having all the sensible qualities of the *lochia*. The odour emitted was exceedingly offensive—so much so as to make the young lady unpleasant to herself and her friends. This state of things continued for more than two months, during which time the patient appeared to enjoy general good health. Her appetite was good; she walked and rode out, and was possessed of a fine flow of spirits. Every day there was more or less discharge of the fœtid character mentioned, until debility, loss of appetite, and symptoms of nervous derangement supervened. To relieve this most unpleasant affection, Valet's preparation of iron was administered with the happiest effect. It was prescribed in the following proportion:

℞. Ferri protocarb. ℈iv.

F. pil. lx.

One three times a day.

By the time the pills were completed, the sallowness of the cheek had given place to a ruddy complexion,—the corporeal strength, which had never been recovered, returned in all its vigour, and the unhealthy discharge had ceased. I may here observe, *en passant*, that I have good reason, from the trials I have given it, to place a large share of confidence in the protocarbonate of iron, as an efficacious remedy in deranged menstruation, of whatever character, as well as in chronic, hysteric, and neuralgic affections.

A few remarks upon this case, and I have done.

*The paroxysms of pain* under which my patient suffered, when I was first called to her, were, no doubt, owing, in a great measure, to the contractions of the uterus, provoked by the fluid distending its coats. What is remarkable is, that they should always have ceased immediately upon the evacuation of the urine. The cause of suppression of this latter, as suggested above, was plainly attributable to mechanical pressure of the menstrual fluid upon the neck of the organ containing it.

The reason why the enlarged uterus was not distinguished at the first visit, was because of the anterior position of the largely distended bladder. With regard to the *qualities of the catamenial discharge*, I would remark, that it was without any very perceptible, certainly without any disagreeable odour,—*entirely free from coagula*,—and in colour and consistence resembling "tar," (though perhaps less dense,) which is referred to as illustrative of its character, in a case reported by Dr. Denman.

The *quantity* contained in the uterus and vagina at the time of the operation, I should suppose to be about half a gallon. What occasioned the hysteritis? Was it the admission of the atmospheric air into the uterine cavity, proving too strong a stimulus for a surface unaccustomed to its action? Or was it a reaction between this and the contained fluid, giving rise to the developement of a deleterious gas, which excited the inflammation? There is no reason to question the *identity* of the disease; that its seat was in the uterus, and not in the peritoneum. In several instances, it appears from the cases reported, death has been occasioned by the division of the hymen; and always, according to the reporters, from *peritoneal* inflammation. How such an event should follow, unless it supervened upon hysteritis, it is difficult to imagine; for I can scarcely conceive that the operation could be so unskilfully performed as to wound the peritoneum through the vagina.

On summing up all the evidence, it would appear that the division of the hymen, though a simple operation in itself, is not to be undertaken without the apprehension of very serious results.

*Chester County, Pa.*, October, 1839.

